# SARS-CoV-2 vaccine effectiveness against infection, symptomatic and severe COVID-19: a systematic review and meta-analysis

**DOI:** 10.1186/s12879-022-07418-y

**Published:** 2022-05-07

**Authors:** Paddy Ssentongo, Anna E. Ssentongo, Navya Voleti, Destin Groff, Ashley Sun, Djibril M. Ba, Jonathan Nunez, Leslie J. Parent, Vernon M. Chinchilli, Catharine I. Paules

**Affiliations:** 1 Department of Public Health Sciences, Penn State College of Medicine and Milton S. Hershey Medical Center, 90 Hope Drive, Suite 2404L, Hershey, PA 17033 USA; 2 Department of Medicine, Penn State College of Medicine and Milton S. Hershey Medical Center, Hershey, PA USA; 3 Department of Surgery, Penn State College of Medicine and Milton S. Hershey Medical Center, Hershey, PA USA; 4 Penn State College of Medicine, Hershey, PA USA; 5 Division of Infectious Diseases and Epidemiology, Department of Medicine, Penn State Hershey College of Medicine and Milton S. Hershey Medical Center, Hershey, PA USA; 6 Division of Infectious Diseases and Epidemiology, Departments of Medicine and Microbiology and Immunology, Penn State College of Medicine, Hershey, PA USA

**Keywords:** Vaccine effectiveness, COVID-19, Waning immunity

## Abstract

**Background:**

The temporal evolution of SARS-CoV-2 vaccine efficacy and effectiveness (VE) against infection, symptomatic, and severe COVID-19 is incompletely defined. The temporal evolution of VE could be dependent on age, vaccine types, variants of the virus, and geographic region. We aimed to conduct a systematic review and meta-analysis of the duration of VE against SARS-CoV-2 infection, symptomatic COVID-19 and severe COVID-19.

**Methods:**

MEDLINE, Scopus, Cochrane Central Register of Controlled Trials, Cochrane Database of Systematic Reviews, the World Health Organization Global Literature on Coronavirus Disease, and CoronaCentral databases were searched and studies were selected. Independent reviewers selected randomized controlled trials and cohort studies with the outcome of interest. Independent reviewers extracted data, and assessed the risk of bias. Meta-analysis was performed with the DerSimonian-Laird random-effects model with Hartung-Knapp-Sidik-Jonkman variance correction. The GRADE (Grading of Recommendations, Assessment, Development and Evaluation) approach was used to assess certainty (quality) of the evidence. Primary outcomes included VE as a function of time against SARS-CoV-2 infection, symptomatic and severe COVID-19.

**Results:**

Eighteen studies were included representing nearly 7 million individuals. VE against all SARS-CoV-2 infections declined from 83% in the first month after completion of the original vaccination series to 22% at 5 months or longer. Similarly, VE against symptomatic COVID-19 declined from 94% in the first month after vaccination to 64% by the fourth month. VE against severe COVID-19 for all ages was high overall, with the level being 90% (95% CI, 87–92%) at five months or longer after being fully vaccinated. VE against severe COVID-19 was lower in individuals ≥ 65 years and those who received Ad26.COV2.S.

**Conclusions:**

VE against SARS-CoV-2 infection and symptomatic COVID-19 waned over time but protection remained high against severe COVID-19. These data can be used to inform public health decisions around the need for booster vaccination.

**Supplementary Information:**

The online version contains supplementary material available at 10.1186/s12879-022-07418-y.

## Background

In an effort to decrease the rate of severe acute respiratory syndrome coronavirus 2 (SARS-CoV-2) infections and cases of severe coronavirus disease 2019 (COVID-19), eight billion doses of COVID-19 vaccines have been deployed worldwide as of November 21st, 2021 [1]. In the United States (US), SARS-CoV-2 vaccines include two messenger RNA vaccines (mRNA) BNT162b2 [2] and mRNA-1273 [3] and one adenovirus vector vaccine (Ad26.COV2.S [4]). As of November 21st, 2021, 59% of the United States and 42% of the world’s population were fully vaccinated against SARS-CoV-2 [1]. In randomized placebo-controlled Phase III trials, the BNT162b2, mRNA-1273, and Ad26.COV2.S vaccines showed 95%, 94%, and 67% efficacy against symptomatic disease due to SARS-CoV-2. However, the temporal evolution of vaccine protection against future SARS-CoV-2 infection, symptomatic, and severe COVID-19 remains poorly understood. As countries around the world face surges of COVID-19 cases, the question of waning immunity and its contribution to new outbreaks must be urgently addressed. In this meta-analysis, our objective was to evaluate the overall, age- and vaccine-specific efficacy/effectiveness (VE) of BNT162b2, mRNA-1273, and Ad26.COV2.S vaccines against SARS-CoV-2 infection, symptomatic, and severe COVID-19 disease over time.

## Methods

Results were reported following Preferred Reporting Items for Systematic Reviews and Meta-analyses (PRISMA) 2020 [5]. This study was deemed exempt by the Penn State Institutional Review Board. The study protocol is provided in Additional file 1: Text S1.

### Data sources and searches

MEDLINE, Scopus, Cochrane Central Register of Controlled Trials, Cochrane Database of Systematic Reviews, the World Health Organization Global Literature on Coronavirus Disease, and CoronaCentral databases were searched from December 2019 to November 2021 for peer-reviewed studies reporting COVID-19 Vaccine effectiveness or efficacy without language restriction. Clinical trial registries through The World Health Organization (WHO) International Clinical Trials Registry Platform search portal (https://trialsearch.who.int/) and conference proceedings were also searched. Reference mining of primary studies was conducted to identify additional literature. The following Medical Subject Headings and keyword search terms were used; “vaccine effectiveness” OR “vaccine efficacy” AND “SARS-CoV-2” OR “COVID-19” OR “severe acute respiratory syndrome coronavirus-2” OR “coronavirus disease 2019”. Full search terms are provided in Additional file 1: Table S1.

### Study selection

Studies were selected according to Participant (P), Intervention (I), Comparator [C], Outcome (O) and Study type (S) [PICOS] criteria [6]:

*Participants* Persons of all ages and sex included in studies that investigated COVID-19 vaccines efficacy or effectiveness.

*Intervention* COVID-19 vaccines (BNT162b2, mRNA-1273, Ad26.COV2.S). We did not include studies that evaluated booster doses because very few studies on efficacy of boosters were available at the time of our meta-analysis.

*Comparison* Unvaccinated cohorts.

*Outcome of interest* Vaccine effectiveness or efficacy (VE) calculated as 100 × (1 − IRR), where IRR (incidence rate ratio) is the ratio of the rate of COVID-19 in the vaccinated group to the corresponding rate in the unvaccinated group. A vaccine’s efficacy is a measure of how well vaccines work in clinical trials. In contrast, vaccine effectiveness is a measure of how well vaccines work in real-world settings outside of a clinical trial [7]. Outcome measures and the definitions of infection and severity of COVID-19 are provided in Additional file 1: Text S2.

*Study type* Randomized clinical trials (RCT) for efficacy and observational studies for effectiveness. Pairs of independent investigators (PS and AES) screened the titles and abstracts of all citations. Studies included by either reviewer were retrieved and independently screened by two investigators (PS and AES).

### Data extraction and quality assessment

A standardized data extraction form was developed and two investigators (PS and AES) worked independently to extract study details. The following information was extracted: year of study publication, country and time frame, type of vaccine; mRNA-1273 (Moderna); BNT162b2 (Pfizer-BioNTech); Ad26.COV2.S (Janssen), inferential statistical test estimates (vaccine effectiveness or efficacy and its 95% confidence intervals), follow-up time after full vaccination (2-doses for mRNA-127 and BNT162b2 and 1 dose for Ad26.COV2.S), study-level descriptive statistics (mean (SD)/ median (IQR) age in years, proportion (%) female, male and obese), follow-up time (days), and definitions of symptomatic, and severe COVID-19. Authors were contacted for missing or incomplete information. The risk of bias of the included RCTs was evaluated with the Cochrane Collaboration’s Risk of Bias 2 tool (Additional file 1: Table S3) [8]. Methodological quality for nonrandomized observational studies was assessed with the Newcastle–Ottawa Scale (NOS) [9]. Based on the NOS criteria, we assigned a maximum of 4 stars for selection, 2 stars for comparability, and 3 stars for exposure and outcome assessment. Studies with fewer than 5 stars were considered low quality; 5 to 7 stars, moderate quality; and more than 7 stars, high quality.

### Grading the quality of evidence

We assessed the quality of evidence using the GRADE (Grading of Recommendations, Assessment, Development and Evaluations) framework using four levels of quality of evidence: very low, low, moderate and high [10].

### Data synthesis and analysis

Statistical analyses were performed with R software version 3.6.2 (R Project for Statistical Computing). R package *ggplot2* was used to display the scatter plots. *Meta* and *Metafor* R packages were used to conduct formal meta-analyses and create forest plots. Descriptive statistics were used to summarize study-level demographics. Meta-analyses were stratified by length of follow-up after full vaccination where continuous time was discretized in months. The DerSimonian-Laird random-effects model with Hartung- Knapp-Sidik-Jonkman variance correction was used to combine VE estimates if the number of studies included in the meta-analysis was greater than three [11–13]. In a situation where VE was reported at more than one time point in a single month of study, a fixed-effects model was utilized to pool the estimate within the study before conducting the random-effect meta-analysis. Results from fixed-effects model were reported for subgroups of 3 studies or fewer.

Heterogeneity between studies was evaluated with the ${I}^{2}$ indicator expressed as percent low (< 25%), moderate (50%), and high (≥ 75%) [14]. Prespecified subgroup analyses were conducted according to age, vaccine type, WHO regions, and study design. Publication bias was quantitatively evaluated with Egger’s linear regression and Begg’s rank test [15, 16] and qualitatively with funnel plots. Two-sided p < 0.05 was deemed statistically significant.

## Results

### Characteristics of studies

**Fig. 1:**
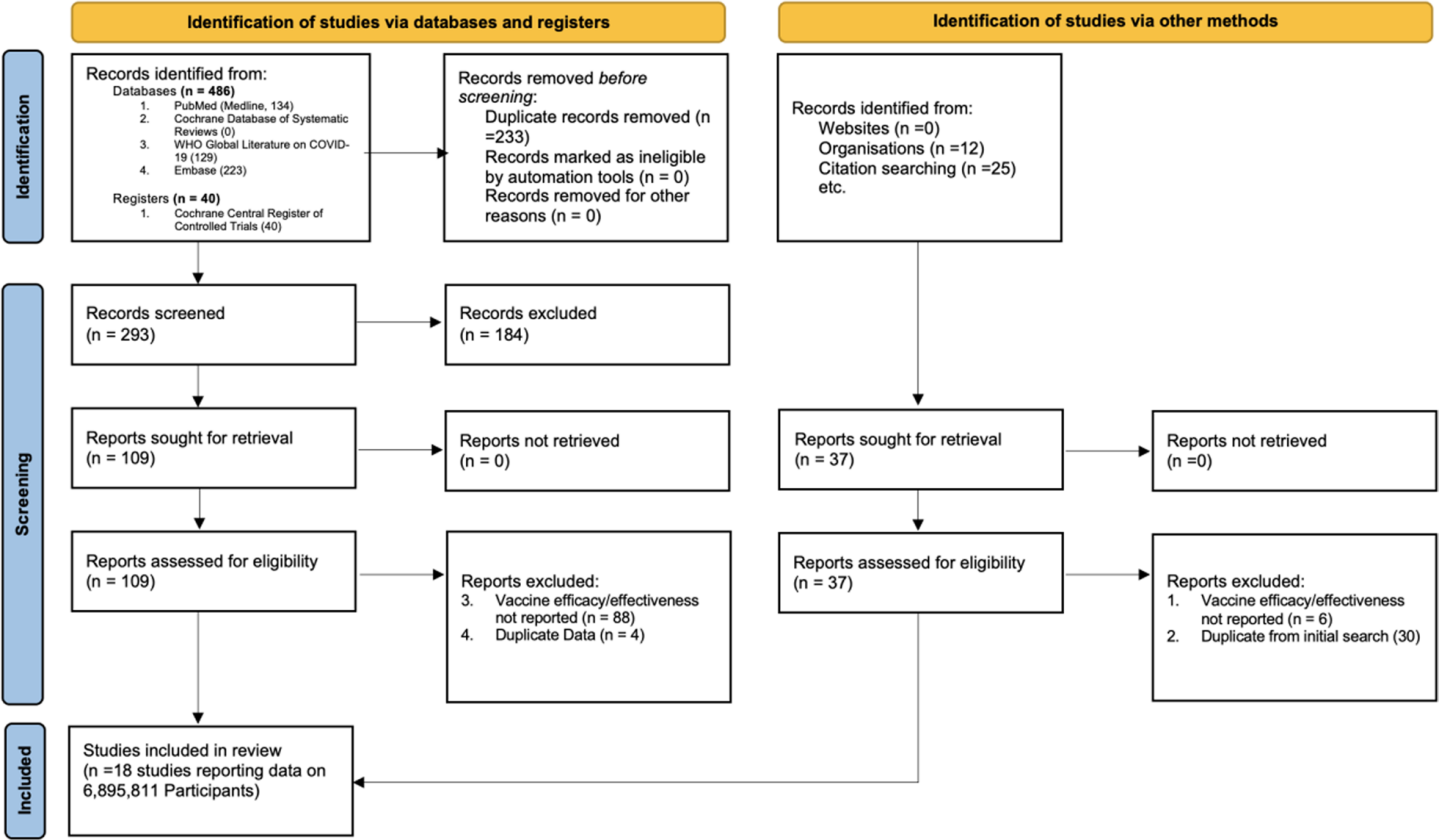
PRISMA 2020 flow diagram for systematic reviews and meta-analysis of vaccine efficacy/effectiveness (VE) against SARS-CoV-2 infection, symptomatic and severe COVID-19

We identified 563 studies. After excluding duplicates, a total of 300 publications were screened for eligibility. After excluding 184 studies based on title and abstract screening, 110 full-text articles were assessed for eligibility (Fig. 1). The final number of articles included was 18 (n = 6,895,811 individuals), consisting of 2,669,506 vaccinated subjects (38.7%) and 4,226,305 unvaccinated controls (61.3%). The studies included 5 randomized controlled trials (RCT), 5 case–control studies, and 8 cohort studies. The median study duration across all studies was 120 days after the second dose of mRNA or the first dose of AD26.COV2.S vaccines (range 0–300 days; interquartile range [IQR] 30–90 days). Eleven studies reported VE starting within 7 days of the second dose of the mRNA vaccine [17–27]. VE was reported starting 14 days after the second dose of mRNA vaccines in two studies (Ali et al. and Tenforde et al. [28, 29]). Three studies reported VE for AD26.COV2.S, one began following patients immediately, and the other two started 14 days after the first dose [4, 24, 30] Seven studies documented VE stratified by age [17–19, 21–23, 31]. The median age across studies was 46 years (range 14–61 years), with 47% of patients being female (range 31–100%). Two studies included adolescents 12–25 years [29], one study included only individuals who were in incarcerated [32], and two studies included only hospitalized patients [28, 30].

The majority of studies included in our meta-analyses only reported VE on one vaccine type. However, Self et al. reported VE for BNT162b2, mRNA-1273, and AD26.COV2.S [30] whereas Paris et al. and Tenford et al. reported VE for both mRNA vaccines (BNT162b2 and mRNA-1273) [28]. VE estimates from mRNA vaccines reported by Self et al. were excluded from our analysis due to overlapping study populations; we included the data they reported on AD26.COV2.S [33]. Details of included studies are provided in Additional file 1: Table S2. Risk-of-bias assesemnt for RCT is provided in Additional file 1: Table S2.

### Temporal evolution of VE against infection

**Fig. 2:**
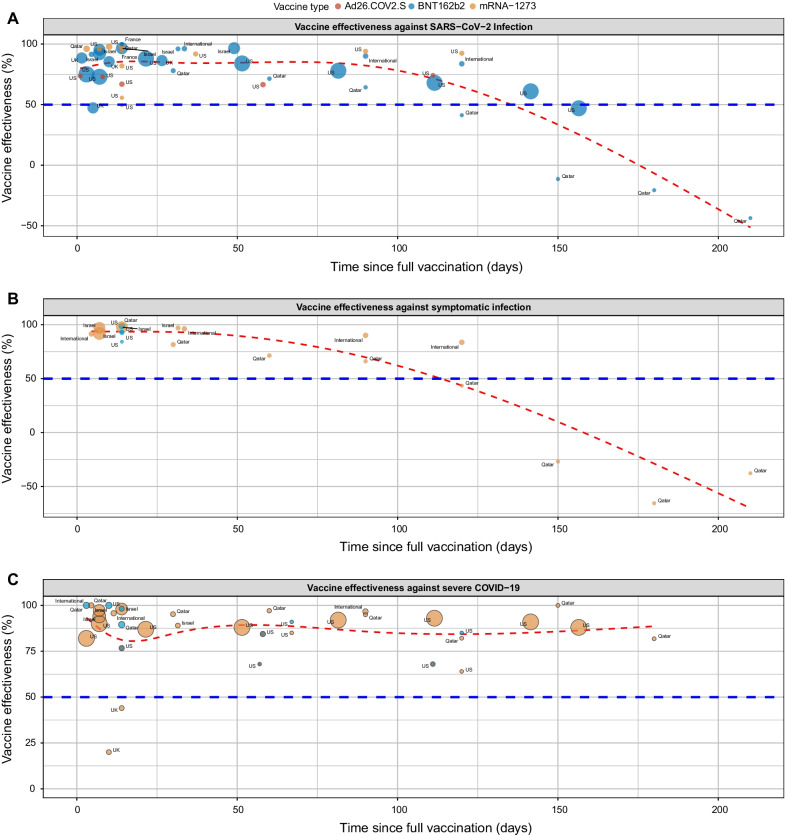
Scatterplot of Vaccine efficacy/effectiveness (VE) % against SARS-CoV-2 infection (**A**), symptomatic COVID-19 (**B**) and severe COVID-19 (**C**) plotted according to time from the second dose for mRNA vaccines and one dose of adenovirus vector-based vaccine. Each circle represents a study, and its size is proportional to the study’s sample size and annotated according to vaccine type. Effectiveness estimates include studies that estimated efficacy. The red line represents the fitted mean VE using the natural cubic spline model. The horizontal blue line represents the 50% protection level stipulated by the WHO [7]

**Fig. 3:**
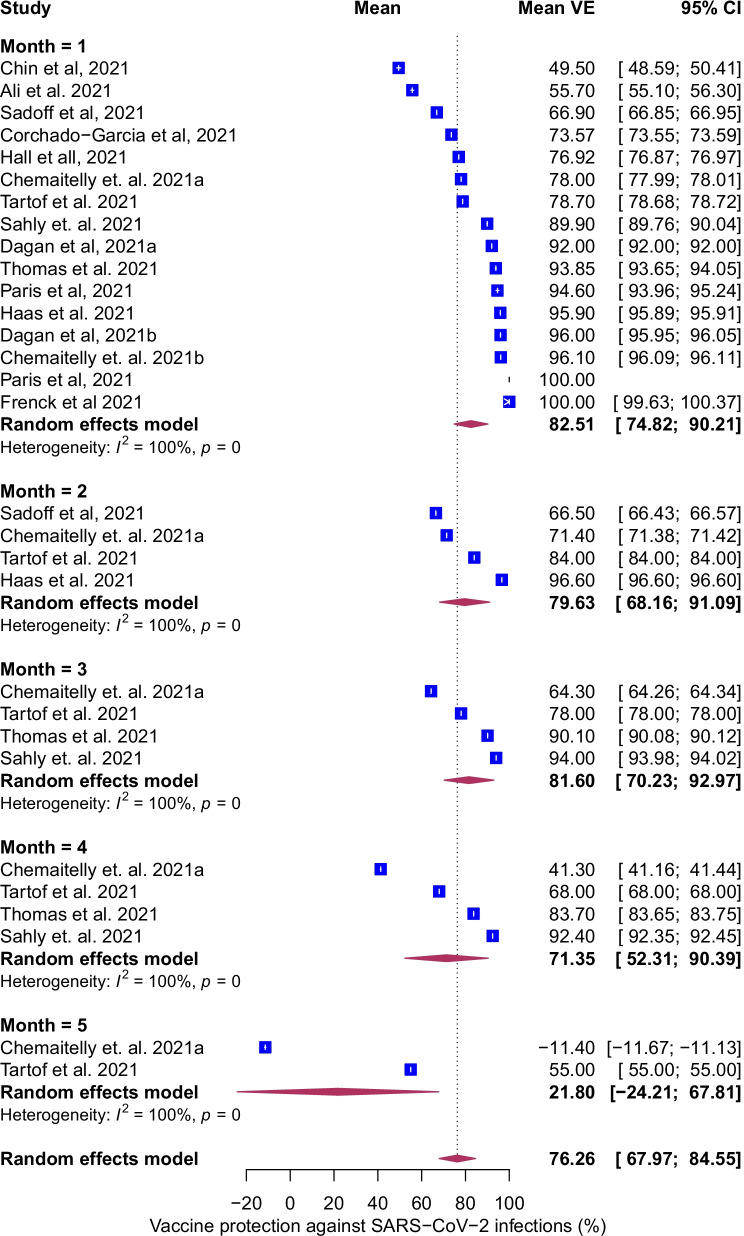
Forest plot of vaccine efficacy/effectiveness (VE) against SARS-CoV-2 infection stratified by months since full vaccination. Mean VE values represent the mean vaccine protection expressed as percentage. Blue squares and their corresponding lines are the point estimates and 95% confidence intervals (95% CI). Maroon diamonds represent the pooled VE estimates for each month (width denotes 95% CI). Effectiveness estimates include studies that estimated efficacy

**Fig. 4:**
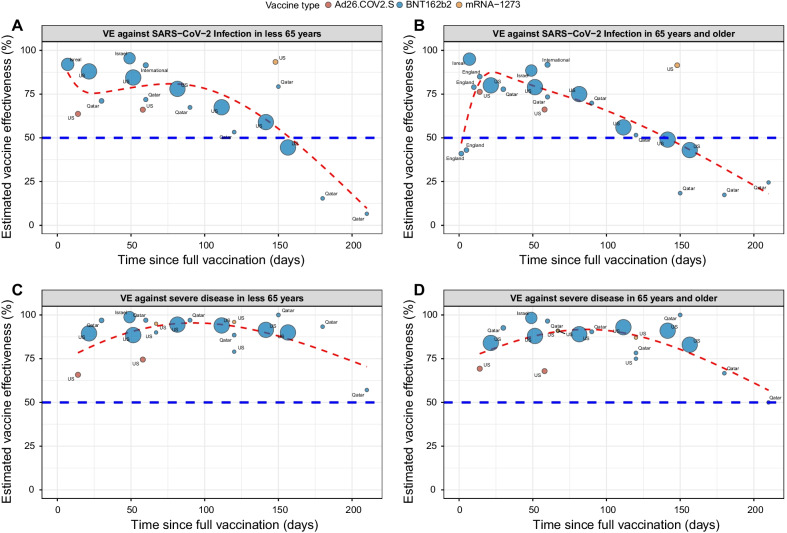
Scatterplot of Vaccine efficacy/effectiveness (VE) against infection and severe disease stratified by age. VE against infection in subjects < 65 years (**A**) ≥ 65 years (**B**), against severe COVID-19 disease in those < 65 years (**C**) and ≥ 65 years (**D**) plotted according to time since full vaccination (14 days after second dose for mRNA vaccines and after one dose of adenovirus vector-based vaccine). Each circle represents a study, and its size is proportional to the study’s sample size and annotated according to vaccine type. The red line represents the fitted mean VE using the natural cubic spline model. The horizontal blue line represents the 50% protection level stipulated by the WHO [7]

To estimate VE against SARS-CoV-2 infection, we examined the association between prior vaccination and the incidence rate of testing positive for SARS-CoV-2 in the vaccinated compared to the unvaccinated group. For all ages and vaccines, pooled mean VE against SARS-CoV-2 infection was 76% (95% CI, 68–85%, *I*^*2*^ = 100%) with variation based on vaccine type, BNT162b2 65% (95% CI, 60–71%), mRNA-1273, 82% (95% CI, 80–84%) and Ad26.COV2.S, 69% (95% CI, 64–74%, Additional file 1: Fig. S1). VE against infection declined sharply after 100 days following full vaccination (Fig. 2A). The mean VE against SARS-CoV-2 infection was 83% (95% CI, 75–90%), 80% (95% CI, 68–91%), 82% (95% CI, 70–93%)**,** 71% (95% CI, 52–90%)**,** and 22% (95% CI, − 24–68%) at 1, 2, 3, 4, and 5 months following vaccination, respectively (Fig. 3). We were unable to conduct vaccine waning subgroup analysis according to vaccine type because only three BNT162b2, one mRNA-1273 and no Ad26.COV2.S studies reported VE estimates against SARS-CoV-2 infection beyond 3 months. We assessed age as an effect modifier in the association between vaccine status and infection. After full vaccination, there was a rapid increase in VE in both age groups which reached a maximum of ~ 88% in the first 30 days (Fig. 4A and B). VE declined more rapidly in individuals ≥ 65 years than < 65 years, beginning at approximately 25 days in individuals ≥ 65 years and 80 days in those < 65 years. By 150 days post vaccination, VE in both age groups declined below 50%.

### Temporal evolution of VE against symptomatic COVID-19

**Fig. 5:**
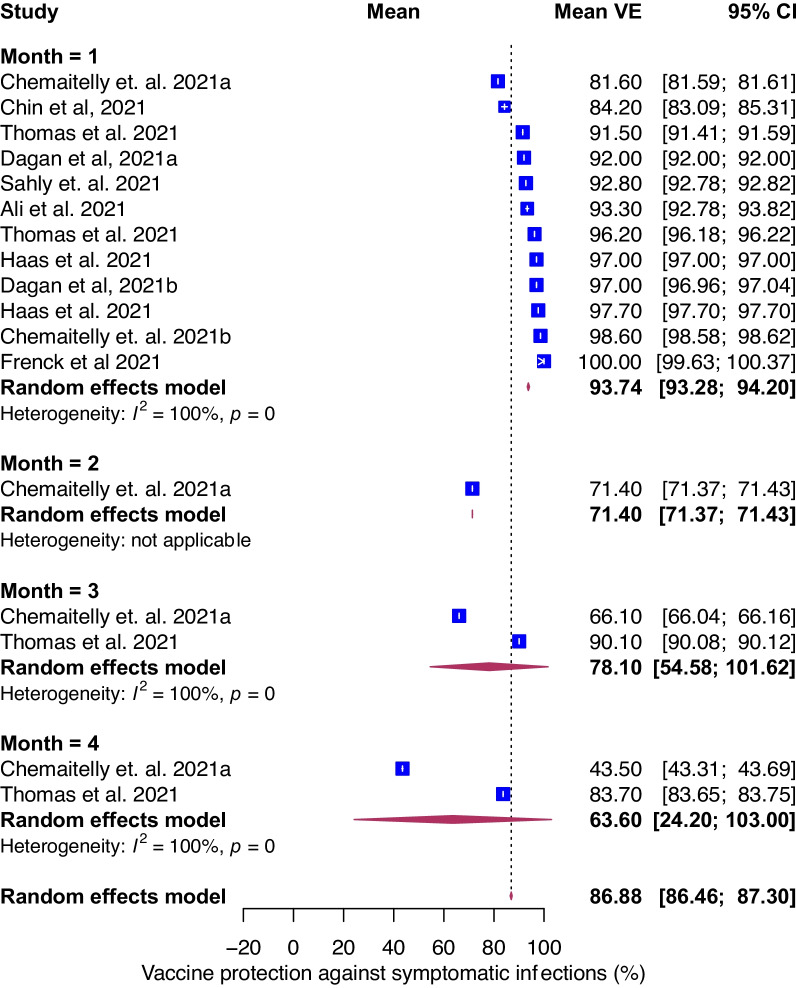
Forest plot of vaccine efficacy/effectiveness (VE) against symptomatic COVID-19 stratified by months since full vaccination. Mean VE values represent the mean vaccine protection expressed as a percentage. Blue squares and their corresponding lines are the point estimates and 95% confidence intervals (95% CI). Maroon diamonds represent the pooled VE estimates for each month (width denotes 95% CI)

Next, we assessed the evolution of VE as function of time for symptomatic COVID-19. For all ages and vaccines, pooled mean VE against symptomatic COVID-19 was 87% (95% CI, 86–87%, *I*^*2*^ = 100%) with variation based on vaccine type; for mRNA-1273 and BNT162b2 VE were 92% (95% CI, 88–96%) and 85% (95% CI, 85–86%), respectively (Additional file 1: Fig. S2). Only one study estimated VE against symptomatic COVID-19 stratified by age for Ad26.COV2.S; VE was 76.3% (95% CI 61.6–86%) for 60 or older and 63.7% (95% CI 53.9–71.6%) for 18–59 at 14 or more days and 66.2% (95% CI 36.7–83.0%) and 66.1% (95% CI 53.3–75.8%) respectively at 28 or more days [4]. The temporal evolution of VE against symptomatic infection was similar to that of overall infection (Fig. 2B). Mean VE declined over time and reached 94% (95% CI, 93–94%), 78% (95% CI, 55–100%), and 64% (95% CI, 24–100%) at 1, 3 and 4-months following vaccination (Fig. 5). Estimates were not reported for month 2 (except for one study) and month 5 (no studies). There were not enough data points to estimate VE against symptomatic infection by age.

### Temporal evolution of VE against severe COVID-19

**Fig. 6:**
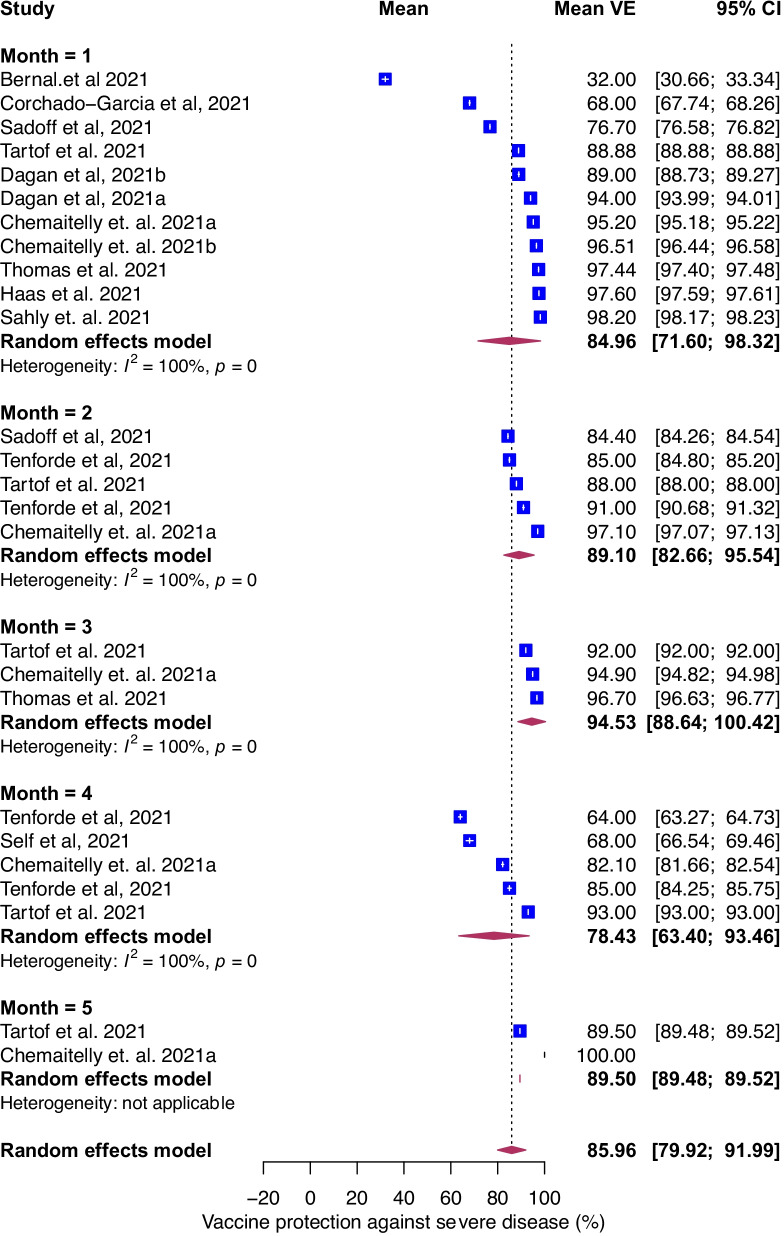
Forest plot of vaccine efficacy/effectiveness against severe COVID-19 stratified by months since full vaccination. Mean VE values represent the mean expressed as a percentage. Blue squares and their corresponding lines are the point estimates and 95% confidence intervals (95% CI). Maroon diamonds represent the pooled VE estimates for each month (width denotes 95% CI)

To estimate the protection of vaccination against severe COVID-19, we examined the association between prior vaccination and severe COVID-19. The definition of severe COVID-19 varied by study (Additional file 1: Table S2). Sensitivity analysis comparing different definitions of severe COVID-19 including critical COVID-19 did not show differences in estimates (data not shown). For all ages and vaccines, the pooled mean VE was 86% (95% CI, 80–92%, *I*^*2*^ = 100%) and the temporal evolution consistently remained robust (> 90%) through 175 days post-vaccination (Fig. 2C). The mean VE was 85% (95% CI, 72–98%), 89% (95% CI, 83–96%), 95% (95% CI, 87–100%), 78% (95% CI, 63–93%), and 90% (95% CI, 89–92%) at 1, 2, 3, 4, and 5 months following vaccination, respectively (Fig. 6). For studies where age was included, we evaluated age as an effect modifier. VE against severe disease reached a maximum of ~ 90% at approximately 60 days post-vaccination and declined to 74% and 62% by day 200 post-vaccination in the < 65 and ≥ 65 years, respectively (Fig. 4C and D). VE against severe COVID-19 was higher for mRNA-1273, 93% (95% CI: 83–100%) than BNT162b2, 87% (95% CI, 79–95% and Ad26.COV2.S 74% (95% CI, 62–87%, Additional file 1: Fig. S3). We were unable to conduct vaccine waning subgroup analysis against severe COVID-19 by vaccine type because only four BNT162b2, two mRNA-1273 and one Ad26.COV2.S studies reported VE estimates against severe COVID-19 beyond 3 months.

### Subgroup and sensitivity analyses

Analyses limited to only efficacy (RCT) trials found a higher VE against SARS-CoV-2 infection compared to effectiveness (non-RCT) studies: 83% (95% CI, 78–89%) vs. 63% (95% CI, 57–68%); symptomatic COVID-19, 93% (95% CI, 90–95%, vs. 83% (95% CI, 82–83%); and severe COVID-19, 91% (95% CI, 79–100%) vs. 85% (95% CI, 78–92%) (Additional file 1: Fig. S4, S5, S6). When compared to studies conducted in Europe and North America, studies conducted in Qatar (representative of Eastern Mediterranean region) showed lower vaccine protection against SARS-CoV-2 infection at 34% (95% CI, 25–44%) and symptomatic COVID-19 at 29% (95% CI, 20–38%), but not for severe COVID-19, at 91% (95% CI, 84–99%) (Additional file 1: Fig. S7, S8, S9). For studies that assessed asymptomatic SARS-CoV-2 infection, VE decreased more rapidly than that of overall infection or symptomatic infection. (Additional file 1: Fig. S10).

No strong evidence of publication bias nor asymmetries in the funnel plots for overall VE against infection, symptomatic and severe COVID-19 was detected. Egger’s test (p = 0.13, p = 0.34, p = 0.54) for publication bias was not significant (Additional file 1: Fig. S11). The overall quality of evidence by the GRADE framework for the VE was graded as ‘moderate’ quality (Additional file 1: Table S2).

## Discussion

In this systematic review and meta-analysis of 18 peer-reviewed studies, which included nearly 7 million individuals, we found evidence of waning immunity against SARS-CoV-2 infection from a high of 83% at one month to 22% at five months or longer after being fully vaccinated. Similar trends were observed for symptomatic COVID-19. VE against SARS-CoV-2 infection declined more rapidly in individuals ≥ age 65 years but was less than 50% in all age groups by month five. VE varied by vaccine type with highest pooled VE among mRNA-1273 recipients. Reassuringly, VE against severe disease remained robust; 90% at five months following vaccination, although protection was lower in older individuals (≥ 65 years) and in those who received Ad26.COV2.S.

The impact of temporal waning of vaccine effectiveness against SARS-CoV-2 infection raises concern that initially effective vaccination strategies will not be sufficient to mitigate the individual and population level effect of COVID-19 long-term. Historically, waning immunity for other infectious diseases has been addressed by administering subsequent doses of vaccine, e.g., booster doses [34]. In line with other highly vaccinated countries, the US Food and Drug Administration (FDA) amended its Emergency Use Authorizations (EUA) for COVID-19 vaccines on November 19, 2021 to allow a single booster dose for all adults age ≥ 18 years and the Centers for Disease control and Prevention has endorsed booster doses for all adults. This approach in well-resourced countries has led to international debate about the implementation of booster vaccine rollout when much of the world’s population has yet to receive a single vaccine dose. Understanding the public health goal of booster vaccinations–prevention of infection versus prevention of symptomatic disease versus prevention of severe COVID-19 outcomes– is crucial to implementing a global strategy moving forward. At the population level, the majority of SARS-CoV-2 infections and arguably more importantly, severe cases, continue to be identified in the unvaccinated or in those unlikely to mount a robust vaccine response [35]. The drivers of population level transmission in regions of variable vaccine uptake have yet to be determined.

Based on our data, VE against SARS-CoV-2 infection and symptomatic COVID-19 are clearly waning and have fallen below the WHO’s minimal criteria of 50% when considering the outcomes of infection and symptomatic disease [7]. If impact on less severe COVID-19 is chosen as a global goal, then booster doses will certainly be needed to attempt and restore higher effectiveness targets. Early data from BNT162b2 vaccination campaigns in Israel and the United Kingdom suggest that a booster dose of COVID-19 vaccine will in the short-term increase vaccine effectiveness against confirmed infection and symptomatic disease [36–38]. The longevity of this protection, beyond a few weeks after vaccination, has yet to be determined and the impact on transmission and utilization of healthcare resources remains unclear.

In terms of severe COVID-19, our data support robust protection that persists over time. A pooled VE of 90% at five months post-vaccination remains well above the WHO’s preferred estimate of 70% and minimal estimate of 50% when considering effectiveness against the outcome of severe disease [7]. We did note an increased risk of severe disease in individuals ≥ 65 years of age and those initially vaccinated with Ad26.COV2.S that warrants further consideration. In this regard, many countries that have implemented booster policies have targeted older individuals first. The impact of this approach is largely unknown, although observational data from Israel suggests that a third dose of BNT162b2 decreased severe COVID-19 in individuals 40 years of age or greater in the short term [39]. However, severe outcomes in immunocompetent individuals who have received BNT162b2 or mRNA-1273 continue to be rare [33] and the side effect profile of a subsequent dose, particularly in fully vaccinated younger individuals at very low risk of COVID-19 complications, has not been fully delineated.

Individuals who received Ad26.COV2.S have lower VE against all outcomes, including pooled VE of 74% against severe COVID-19. Unpublished data presented to the Advisory Committee for Immunization Practices on October 21, 2021 suggested that a booster dose of Ad26.COV2.S given two months after the initial immunization increased VE in the short term to 100% against severe disease [40].

Several important limitations of this meta-analysis should be considered when utilizing the data to influence public health policies. There is high between-study variation in the included estimates. Several factors may have contributed to this variation. First, study designs ranged from high-quality randomized controlled clinical trials to lesser quality cohort and test-negative case–control studies. When considering only the highest quality studies, VE was 83%, 93% and 91% for infection, symptomatic, and severe COVID-19, respectively. However, while the RCTs had higher estimates and controlled trials are generally considered to have a higher level of evidence, some of the RCTs included had short follow-up periods, very different participant groups (teenagers), and potential bias (baseline comparison groups not similar) as shown from Additional file 1: Table S2. Second, study populations varied geographically according to the WHO regions and included the Americas (United States, South America), Europe (United Kingdom, Israel), Africa (South Africa), and Eastern Mediterranean Region (Qatar). Random-effects models were adopted to control for the possible difference in effect estimates by region. However, potential differences in study demographics, rates of post-infection immunity, and political and social interventions exercised by these distinct geographic regions to control the pandemic could have introduced variations in VE. Third, we chose to focus our analysis on only mRNA-1273, BNT162b2, and Ad26.COV2.S. Therefore, our findings are not generalizable to other globally-available vaccines. Fourth, we could not conduct time-varying estimates stratified by the vaccine type due to small number of studies. Fifth, our analysis was temporally limited to 5–6 months after full vaccination as dictated by data availability. Impact of waning VE on all outcomes beyond the time frame of this study remains unknown. Lastly, our analysis was conducted before the emergence of the Omicron variant, which is associated with lower VE than the Delta variant [41], suggesting potential variability in VE as future variants emerge.

## Conclusions

There is ongoing international debate about the necessity of booster dosing as many highly vaccinated countries experience COVID-19 surges. Our data clearly illustrate the temporal waning of VE against SARS-COV-2 infection and symptomatic illness with preservation of VE in most circumstances against severe illness. The need for booster vaccines doses should be considered in the context of clearly outlined international public health goals for vaccine effectiveness outcomes [36]. In areas where booster doses are implemented our data support targeting individuals who received Ad26.COV2.S and those ≥ 65 years for initial vaccine rollout.

## Supplementary Information

**Additional file 1:** Supplmentary Tables and Figures.

## Data Availability

To facilitate replication of these findings, the additional material includes the full dataset. R code and data to reproduce the results in the present manuscript are archived at GitHub. R code and data to reproduce the results in the present manuscript are archived at GitHub (https://github.com/ssentongojeddy/COVID19-Immunity-Waning).

## References

[1] Mathieu E, Ritchie H, Ortiz-Ospina E, Roser M, Hasell J, Appel C, Giattino C, Rodés-Guirao L (2021). A global database of COVID-19 vaccinations. Nat Human Behav.

[2] Polack FP, Thomas SJ, Kitchin N, Absalon J, Gurtman A, Lockhart S, Perez JL, Marc GP, Moreira ED, Zerbini C (2020). Safety and efficacy of the BNT162b2 mRNA Covid-19 vaccine. New Engl J Med.

[3] Baden LR, El Sahly HM, Essink B, Kotloff K, Frey S, Novak R, Diemert D, Spector SA, Rouphael N, Creech CB (2021). Efficacy and safety of the mRNA-1273 SARS-CoV-2 vaccine. N Engl J Med.

[4] Sadoff J, Gray G, Vandebosch A, Cárdenas V, Shukarev G, Grinsztejn B, Goepfert PA, Truyers C, Fennema H, Spiessens B (2021). Safety and efficacy of single-dose Ad26. COV2. S vaccine against COVID-19. New Engl J Med.

[5] Page MJ, McKenzie JE, Bossuyt PM, Boutron I, Hoffmann TC, Mulrow CD, Shamseer L, Tetzlaff JM, Akl EA, Brennan SE (2021). The PRISMA 2020 statement: an updated guideline for reporting systematic reviews. BMJ.

[6] Methley AM, Campbell S, Chew-Graham C, McNally R, Cheraghi-Sohi S (2014). PICO, PICOS and SPIDER: a comparison study of specificity and sensitivity in three search tools for qualitative systematic reviews. BMC Health Serv Res.

[7] Vaccine efficacy, effectiveness and protection. https://www.who.int/news-room/feature-stories/detail/vaccine-efficacy-effectiveness-and-protection.

[8] Higgins J, Altman DG. Assessing risk of bias in included studies. 2008.

[9] Wells GA, Shea B, O’Connell D, Peterson J, Welch V, Losos M, Tugwell P. The Newcastle-Ottawa Scale (NOS) for assessing the quality of nonrandomised studies in meta-analyses. In*.*: Oxford; 2000.

[10] Guyatt GH, Oxman AD, Vist GE, Kunz R, Falck-Ytter Y, Alonso-Coello P, Schünemann HJ (2008). GRADE: an emerging consensus on rating quality of evidence and strength of recommendations. BMJ.

[11] DerSimonian R, Kacker R (2007). Random-effects model for meta-analysis of clinical trials: an update. Contemp Clin Trials.

[12] Sidik K, Jonkman JN (2006). Robust variance estimation for random effects meta-analysis. Comput Stat Data Anal.

[13] IntHout J, Ioannidis JP, Borm GF (2014). The Hartung-Knapp-Sidik-Jonkman method for random effects meta-analysis is straightforward and considerably outperforms the standard DerSimonian-Laird method. BMC Med Res Methodol.

[14] Higgins JP, Thompson SG, Deeks JJ, Altman DG (2003). Measuring inconsistency in meta-analyses. BMJ.

[15] Egger M, Smith GD, Schneider M, Minder C (1997). Bias in meta-analysis detected by a simple, graphical test. BMJ.

[16] Begg CB, Mazumdar M (1994). Operating characteristics of a rank correlation test for publication bias. Biometrics.

[17] Haas EJ, Angulo FJ, McLaughlin JM, Anis E, Singer SR, Khan F, Brooks N, Smaja M, Mircus G, Pan K (2021). Impact and effectiveness of mRNA BNT162b2 vaccine against SARS-CoV-2 infections and COVID-19 cases, hospitalisations, and deaths following a nationwide vaccination campaign in Israel: an observational study using national surveillance data. The Lancet.

[18] Tartof SY, Slezak JM, Fischer H, Hong V, Ackerson BK, Ranasinghe ON, Frankland TB, Ogun OA, Zamparo JM, Gray S (2021). Effectiveness of mRNA BNT162b2 COVID-19 vaccine up to 6 months in a large integrated health system in the USA: a retrospective cohort study. The Lancet.

[19] El Sahly HM, Baden LR, Essink B, Doblecki-Lewis S, Martin JM, Anderson EJ, Campbell TB, Clark J, Jackson LA, Fichtenbaum CJ (2021). Efficacy of the mRNA-1273 SARS-CoV-2 vaccine at completion of blinded phase. New Engl J Med.

[20] Chemaitelly H, Yassine HM, Benslimane FM, Al Khatib HA, Tang P, Hasan MR, Malek JA, Coyle P, Ayoub HH, Al Kanaani Z (2021). mRNA-1273 COVID-19 vaccine effectiveness against the B 11 7 and B 1351 variants and severe COVID-19 disease in Qatar. Nat Med.

[21] Thomas SJ, Moreira ED, Kitchin N, Absalon J, Gurtman A, Lockhart S, Perez JL, Pérez Marc G, Polack FP, Zerbini C (2021). Safety and efficacy of the BNT162b2 mRNA Covid-19 vaccine through 6 months. New Engl J Med.

[22] Chemaitelly H, Tang P, Hasan MR, AlMukdad S, Yassine HM, Benslimane FM, Al Khatib HA, Coyle P, Ayoub HH, Al Kanaani Z (2021). Waning of BNT162b2 vaccine protection against SARS-CoV-2 infection in Qatar. New Engl J Med.

[23] Dagan N, Barda N, Kepten E, Miron O, Perchik S, Katz MA, Hernán MA, Lipsitch M, Reis B, Balicer RD (2021). BNT162b2 mRNA Covid-19 vaccine in a nationwide mass vaccination setting. N Engl J Med.

[24] Corchado-Garcia J, Hughes T, Cristea-Platon T, Lenehan P, Pawlowski C, Bade S, O'Horo JC, Gores GJ, Williams AW, Badley AD. Real-world effectiveness of Ad26. COV2. S adenoviral vector vaccine for COVID-19. 2021. 10.1001/jamanetworkopen.2021.32540 PMC8564583 34726743

[25] Hall VJ, Foulkes S, Saei A, Andrews N, Oguti B, Charlett A, Wellington E, Stowe J, Gillson N, Atti A (2021). COVID-19 vaccine coverage in health-care workers in England and effectiveness of BNT162b2 mRNA vaccine against infection (SIREN): a prospective, multicentre, cohort study. The Lancet.

[26] Dagan N, Barda N, Biron-Shental T, Makov-Assif M, Key C, Kohane IS, Hernán MA, Lipsitch M, Hernandez-Diaz S, Reis BY (2021). Effectiveness of the BNT162b2 mRNA COVID-19 vaccine in pregnancy. Nat Med.

[27] Bernal JL, Andrews N, Gower C, Robertson C, Stowe J, Tessier E, Simmons R, Cottrell S, Roberts R, O’Doherty M. Effectiveness of the Pfizer-BioNTech and Oxford-AstraZeneca vaccines on covid-19 related symptoms, hospital admissions, and mortality in older adults in England: test negative case-control study. BMJ. 2021; 373. 10.1136/bmj.n1088 PMC8116636 33985964

[28] Tenforde MW, Self WH, Adams K, Gaglani M, Ginde AA, McNeal T, Ghamande S, Douin DJ, Talbot HK, Casey JD (2021). Association between mRNA vaccination and COVID-19 hospitalization and disease severity. JAMA.

[29] Ali K, Berman G, Zhou H, Deng W, Faughnan V, Coronado-Voges M, Ding B, Dooley J, Girard B, Hillebrand W (2021). Evaluation of mRNA-1273 SARS-CoV-2 vaccine in adolescents. New Engl J Med.

[30] Self WH, Tenforde MW, Rhoads JP, Gaglani M, Ginde AA, Douin DJ, Olson SM, Talbot HK, Casey JD, Mohr NM (2021). Comparative effectiveness of Moderna, Pfizer-BioNTech, and Janssen (Johnson & Johnson) vaccines in preventing COVID-19 hospitalizations among adults without immunocompromising conditions—United States, March–August 2021. Morb Mortal Wkly Rep.

[31] Frenck RW, Klein NP, Kitchin N, Gurtman A, Absalon J, Lockhart S, Perez JL, Walter EB, Senders S, Bailey R (2021). Safety, immunogenicity, and efficacy of the BNT162b2 Covid-19 vaccine in adolescents. New Engl J Med.

[32] Chin ET, Leidner D, Zhang Y, Long E, Prince L, Li Y, Andrews JR, Studdert DM, Goldhaber-Fiebert JD, Salomon JA (2021). Effectiveness of the mRNA-1273 vaccine during a SARS-CoV-2 delta outbreak in a prison. New Engl J Med.

[33] Paris C, Perrin S, Hamonic S, Bourget B, Roué C, Brassard O, Tadié E, Gicquel V, Bénézit F, Thibault V (2021). Effectiveness of mRNA-BNT162b2, mRNA-1273, and ChAdOx1 nCoV-19 vaccines against COVID-19 in healthcare workers: an observational study using surveillance data. Clin Microbiol Infect.

[34] Hughes SL, Bolotin S, Khan S, Li Y, Johnson C, Friedman L, Tricco AC, Hahné SJ, Heffernan JM, Dabbagh A (2020). The effect of time since measles vaccination and age at first dose on measles vaccine effectiveness–a systematic review. Vaccine.

[35] Scobie HM, Johnson AG, Suthar AB, Severson R, Alden NB, Balter S, Bertolino D, Blythe D, Brady S, Cadwell B (2021). Monitoring incidence of covid-19 cases, hospitalizations, and deaths, by vaccination status—13 US jurisdictions, April 4–July 17, 2021. Morb Mortal Wkly Rep.

[36] Bar-On YM, Goldberg Y, Mandel M, Bodenheimer O, Freedman L, Kalkstein N, Mizrahi B, Alroy-Preis S, Ash N, Milo R (2021). Protection of BNT162b2 vaccine booster against COVID-19 in Israel. N Engl J Med.

[37] Patalon T, Gazit S, Pitzer VE, Prunas O, Warren JL, Weinberger DM (2021). Short term reduction in the odds of testing positive for SARS-CoV-2; a comparison between two doses and three doses of the BNT162b2 vaccine. medRxiv.

[38] Andrews N, Stowe J, Kirsebom F, Gower C, Ramsay M, Bernal JL (2021). Effectiveness of BNT162b2 (Comirnaty, Pfizer-BioNTech) COVID-19 booster vaccine against covid-19 related symptoms in England: test negative case-control study. Medrxiv.

[39] Barda N, Dagan N, Cohen C, Hernán MA, Lipsitch M, Kohane IS, Reis BY, Balicer RD (2021). Effectiveness of a third dose of the BNT162b2 mRNA COVID-19 vaccine for preventing severe outcomes in Israel: an observational study. The Lancet.

[40] Heaton PM, Douoguih M. Booster dose of Janssen COVID-19 Vaccine (Ad26. COV2. S) following primary vaccination. 2021.

[41] Andrews N, Stowe J, Kirsebom F, Toffa S, Rickeard T, Gallagher E, Gower C, Kall M, Groves N, O’Connell A-M (2022). COVID-19 vaccine effectiveness against the omicron (B. 1.1. 529) variant. New Engl J Med.

