# Supplementary material for: SARS-CoV-2 vaccine effectiveness against infection, symptomatic and severe COVID-19: a systematic review and meta-analysis
Source: BMC Infect Dis. 2022 May 7;22:439. doi: 10.1186/s12879-022-07418-y (PMC9077344; doi:10.1186/s12879-022-07418-y)
Supplement: Supplementary file 1 — Additional file 1: Supplmentary Tables and Figures. [file 12879_2022_7418_MOESM1_ESM.docx]

# Supplement to:

# **SARS-CoV-2 Vaccine Effectiveness Against Infection, Symptomatic and Severe COVID-19: A Systematic Review and Meta-analysis**

SUPPLEMENTARY APPENDIX

**Text S1: Study protocol**

**Text S2: Outcome Measures and Definitions of infection and illness outcomes and severity.**

**Table S1: Search terms.**

**Table S2: Characteristics of Studies.**

**Table S2: Cochrane-risk-of-bias tool for randomized trials.**

**Figure S1:** **Forest plot of vaccine efficacy/effectiveness (VE) against SARS-CoV-2 infection stratified by vaccine types.**

**Figure S2:** **Forest plot of vaccine efficacy/effectiveness (VE) against symptomatic COVID-19 stratified by vaccine types.**

**Figure S3:** **Forest plot of vaccine efficacy/effectiveness (VE) against severe COVID-19 stratified by vaccine types.**

**Figure S**4**:** **Forest plot of vaccine efficacy/effectiveness (VE) against SARS-CoV-2 infection stratified by RCT (efficacy trials) vs. non-RCT (effectiveness studies).**

**Figure S5:** **Forest plot of vaccine efficacy/effectiveness (VE) against symptomatic COVID-19 stratified by RCT (efficacy trials) vs. non-RCT (effectiveness studies).**

**Figure S6:** **Forest plot of vaccine efficacy/effectiveness (VE) against severe COVID-19 stratified by RCT (efficacy trials) vs. non-RCT (effectiveness studies).**

**Figure S7:** **Forest plot of vaccine efficacy/effectiveness (VE) against SARS-CoV-2 infection stratified by country of the study.**

**Figure S8: Forest plot of vaccine efficacy/effectiveness (VE) against symptomatic COVID-19 stratified by country of the study.**

**Figure S9: Forest plot of vaccine efficacy/effectiveness (VE) against severe COVID-19 stratified by country of the study.**

**Figure S10: Figure S11: Scatterplot and trend of vaccine efficacy/effectiveness (VE, %) against asymptomatic SARS-CoV-2 infection and critical COVID-19 .**

**Figure S11: Funnel plot for the studies included in the meta-analysis.**

**Text S1: Study protocol**

**Background**

The temporal evolution of SARS-CoV-2 vaccine protection against future infection and severe disease remains poorly understood. The Coronavirus disease 2019 (COVID-19) pandemic was declared a worldwide pandemic on March 11, 2020^1^ . To decrease the rate of infection, rate of severe infection, and other medical, financial, and societal consequences of the disease, two messenger RNA (mRNA vaccines, BNT162b2^1,^ and mRNA-1273^2^) adenovirus vector vaccine Ad26.COV2.S ^3^ COVID-19 vaccines were launched massively in the United States in late December 2020 and early 2021, respectively. In randomized placebo-controlled Phase III trials, the BNT162b2, mRNA-1273, and Ad26.COV2.S showed 95%, 94%, and 67% efficacy against symptomatic and severe COVID-19 disease due to SARS-CoV-2. However, the evolution of the benefits of these vaccines for preventing SARS-CoV-2 infection and severe COVID-19 disease are not systematically characterized. With over 7 billion vaccine doses administered (per the WHO COVID-19 Dashboard) and continued strain on healthcare systems, it is essential to evaluate the continued efficacy of the BNT162b2 vaccine over time.

As vaccination was scaled up, the United States and other parts of the world experienced new variants. Global transmission of the delta variant in June and July 2021 raised concerns about the reduced effectiveness of various COVID-19 vaccines against SARS-CoV-2 infections. However, two doses of mRNA vaccines have shown robust amounts of neutralizing antibodies against all variants of concern evaluated to date[1, 2] and high effectiveness against COVID-19 in various settings.[3-6] These studies even differentiated the potential waning immunity and effectiveness against the effect of SARS-CoV-2 infections amongst the delta variant. In one retrospective cohort study, vaccine effectiveness waned in both non-delta (97% to 67%) and delta infections (93% to 53%) from one month to four months after vaccination.[7] This distinction is essential to inform the need for booster doses and establish the antigenic composition of future vaccines.

**Objectives**

To evaluate the overall vaccine and age-specific vaccine effectiveness of COVID-19 against SARS-CoV-2 infections and severe COVID-19-19 overtime since vaccination in various settings globally through a systematic review and meta-analysis.

**Study selection**

Studies will be selected according to Participant (P) Intervention (I) Comparator [C], Outcome (O) Study type (S) [PICOS] criteria:[8]

**Participants:** Persons of all ages and sex.

**Intervention**: COVID-19 vaccines (BNT162b2, mRNA-1273, Ad26.COV2.S).

**Comparison:** Unvaccinated cohorts.

**Outcome of interest**: Vaccine effectiveness or efficacy (VE) will be calculated as 100 × (1 – IRR), where IRR (incidence rate ratio) is the ratio of the rate of COVID-19 in the vaccinated group to the corresponding rate in the unvaccinated group. A vaccine’s **efficacy** is a measure of how well vaccines work in clinical trials. In contrast, vaccine **effectiveness** is a measure of how well vaccines work in the real-world settings, outside of a clinical trial.[9]

**Study type:** Randomized clinical trials (RCT) for efficacy and observational studies for effectiveness. Pairs of independent investigators (PS and AES) will screen the titles, abstracts and full text.

**Database searches**

The databases to be searched include:

MEDLINE, Scopus, Cochrane Central Register of Controlled Trials, Cochrane Database of Systematic Reviews, the World Health Organization Global Literature on Coronavirus Disease, CoronaCentral databases, clinical trial registries and conference proceedings. Date of search will be limited between December 2019 to November 2021.

**Search Terms**

Medical Subject Headings (MeSH) with various combinations of “vaccine effectiveness” OR “vaccine efficacy” AND “SARS-CoV-2” OR “COVID-19” OR “severe acute respiratory syndrome coronavirus-2” OR “coronavirus disease 2019”.

**Full-text screening and data extraction**

PS. and AES will work independently to extract study details. We will extract the following information year of study publication, country and time frame, type of vaccine; mRNA-1273 (Moderna); BNT162b2 (Pfizer-BioNTech); Ad26.COV2.S (Janssen), inferential statistical test estimates (vaccine effectiveness or efficacy and their 95% confidence intervals), follow-up time after full vaccination (2-doses for mRNA-127 and BNT162b2 and 1 dose for Ad26.COV2.S), study-level descriptive statistics (mean (SD)/ median (IQR) age in years, proportion (%) female, male and obese), follow-up time (days), and definitions of symptomatic, and severe COVID-19. The risk of bias of the included RCTs will be evaluated with the Cochrane Collaboration’s Risk of Bias 2 tool.[10] Methodological quality for nonrandomized observational studies will be assessed with the Newcastle-Ottawa Scale (NOS).[11]

**Data Analysis**

Statistical analyses will be carried out with R software version 3.6.2 (R Project for Statistical Computing). *Meta* and *Metafor* R packages will be used to conduct formal meta-analyses and create forest plots. Meta-analyses will be stratified by length of follow-up after full vaccination. The DerSimonian-Laird random-effects model with Hartung- Knapp-Sidik-Jonkman variance correction will be employed to combine the VE estimate.[12-14]

Between studies heterogeneity will be evaluated with the $I^{2}$ indicator expressed as percent low (25%), moderate (50%), and high (75%).[15] Subgroup analyses will be conducted according to age, vaccine type, World Health Organization regions, and study design. Publication bias will be quantitatively evaluated with Egger’s linear regression and Begg’s rank test[16, 17] and qualitatively with funnel plots.

**Text S2: Definitions of infection and illness outcomes and severity**

**Outcome Measures**

The primary outcomes were time-varying VE against SARS-CoV-2 infection, symptomatic and severe COVID-19. We conducted a secondary subgroup analysis of VE against all primary outcomes by age in persons <65 and ≥ 65 years. Some studies reported VE in more than two age categories. For example, VE for 16-25 years, 26-40 years, 41-64 years, 65 years and older. In these studies, a weighted average of VE of all age categories less than 65 years was performed to obtain a single estimate for the category of <65 years.

**Definition of any SARS-CoV-2 infection:**

The details of the definition of the SARS-CoV-2 infection by each study is provided in Table 1. SARS-CoV-2 infection was defined as

Positive testing by virologic test (i.e., a nucleic acid amplification particularly real-time reverse transcription polymerase chain reaction (RT-PCR) or antigen tests on nasopharyngeal or nasal swab specimens

**Definition of asymptomatic SARS-CoV-2 infection:**

All studies defined asymptomatic infection as a positive test without the presence of any symptoms.

**Definition of mild symptomatic COVID-19:**

**Table 1 lists** the definitions of symptomatic infection provided by all studies. Symptoms included fever, cough, sore throat, malaise, headache, muscle pain, nausea, vomiting, diarrhea, and loss of taste or smell, without shortness of breath or dyspnea.[18]

**Definition of severe COVID-19:**

This definition includes a positive test by standard RT-PCR assay or antigen in addition to symptoms suggestive of severe systemic illness with COVID-19, which could include any symptom of moderate illness or shortness of breath at rest, or respiratory distress. Clinical signs indicative of severe systemic illness with COVID-19, such as respiratory rate ≥ 30 per minute, heart rate ≥ 125 per minute, SpO2 ≤ 93% on room air at sea level or PaO2/FiO2 < 300. No criteria for Critical Severity. Sahly and colleagues combined critical and severe infection into one VE estimate, which we included.

**Definition of critical COVID-19:**

- Positive testing by virologic test
- Evidence of critical illness, defined by at least one of the following;

1. Respiratory failure (endotracheal intubation and mechanical ventilation, oxygen delivered by high-flow nasal cannula, noninvasive positive pressure ventilation, ECMO, or clinical diagnosis of respiratory failure), shock, defined as systolic blood pressure <90 mm Hg, or diastolic blood pressure < 60mm Hg or requiring vasopressors) or multi-organ dysfunction/ failure.
2. Shock (defined by systolic blood pressure < 90 mm Hg, or diastolic blood pressure < 60 mm Hg or requiring vasopressors).
3. Multiorgan disfunction/failure.

**Table S1: Search terms**

| **Database** | **Search Terms** |
| --- | --- |
| **-**MEDLINE  -Scopus,  -Cochrane Central Register of Controlled Trials, -Cochrane Database of Systematic Reviews,  -The World Health Organization Global Literature on Coronavirus Disease,  -CoronaCentral databases and -Clinical trial registries. | 1. “ vaccine effectiveness”).mp. [mp=title, abstract, original title, name of substance word, subject heading word, keyword heading word, protocol supplementary  concept, rare disease supplementary concept, unique identifier]  2. (“vaccine efficacy”).mp. [mp=title,  abstract, original title, name of substance word, subject heading word, keyword heading word,  protocol supplementary concept, rare disease supplementary concept, unique identifier]  3. (“SARS-CoV-2”).mp. [mp=title,  abstract, original title, name of substance word, subject heading word, keyword heading word,  protocol supplementary concept, rare disease supplementary concept, unique identifier]  4. (“COVID-19”).mp. [mp=title,  abstract, original title, name of substance word, subject heading word, keyword heading word,  protocol supplementary concept, rare disease supplementary concept, unique identifier]  5. (“severe acute respiratory syndrome coronavirus-2” ).mp. [mp=title,  abstract, original title, name of substance word, subject heading word, keyword heading word,  protocol supplementary concept, rare disease supplementary concept, unique identifier]  6. (“coronavirus disease 2019”).mp. [mp=title,  abstract, original title, name of substance word, subject heading word, keyword heading word,  protocol supplementary concept, rare disease supplementary concept, unique identifier]  7. 1 OR 2  8. 3 OR 4 OR 5 OR 6  9. 7 AND 8 |

**Table S2: Characteristics of Studies included in the meta-analysis**¶.

| **Study** | **Thomas**  **et al** | **Haas et al** | **Tartof et al** | **Ali et al** | **Chemaitelly et al** | **Sahly**  **et al** | **Chemaitelly et al** | **Self**  **et al** | **Hall**  **et al** | **Dagan et al** | **Bernal et al** | **Tenforde**  **et al** | **Corchado-Garcia et al** | **Dagan et al** | **Sadoff et al** | **Frenck et al** | **Paris et al** | **Chin et al** |
| --- | --- | --- | --- | --- | --- | --- | --- | --- | --- | --- | --- | --- | --- | --- | --- | --- | --- | --- |
| **Date** | Sept 2021 | May 2021 | Oct 2021 | Aug  2021 | Oct  2021 | Sept  2021 | July  2021 | Sept  2021 | May 2021 | Sept 2021 | May 2021 | Nov  2021 | Nov  2021 | Apr 2021 | June  2021 | July  2021 | July  2021 | Oct 2021 |
| **Country** | International | Israel | US | US | Qatar | US | Qatar | US | UK | Israel | UK | US | US | Israel | International | US | France | US |
| **Study design** | RCT | Cohort | Cohort | RCT | Case-control | RCT | Case-control | Case-control | Cohort | Cohort | Case-control | Case-control | Cohort | Cohort | RCT | RCT | Cohort | Cohort |
| **Vaccines** | BNT162b2 | BNT162b2 | BNT162b2 | mRNA-1273 | BNT162b2 | mRNA-1273 | mRNA-1273 | Ad26.COV2.S | BNT162b2 | BNT162b2 | BNT162b2 | mRNA-1273/ BNT162b2 | Ad26.COV2.S | BNT162b2 | Ad26.COV2.S | BNT162b2 | mRNA-1273/ BNT162b2 | mRNA-1273 |
| **Vaccinated (N)** | 22026 | 470000 | 1043289 | 2486 | 12901 | 15209 | 52442 | 113 | 396318 | 10861 | 12872 | 314 | 8698 | 596618 | 21895 | 1131 | 2042 | 291 |
| **Placebo (N)** | 22021 | 180000 | 2290189 | 1240 | 218925 | 15206 | 52442 | 1463 | 710587 | 10861 | 11758 | 1669 | 86495 | 596618 | 21888 | 1129 | 3573 | 241 |
| **% Male** | 51 | 50 | 48 | 51 | 69 | 53 | 47 | 59 | 16 |  | 45 | 51 | 50 | 50 | 55 | 50 |  |  |
| **% White** | 82 | 100 | 32 | 84 |  | 79 |  | 51 | 88 |  | 87 |  | 90 |  | 59 | 86 | 37 | 23 |
| **% Black** | 10 | 0 | 8 | 3 |  | 10 |  | 23 | 2 |  | 1 |  | 3 |  | 19 | 9 |  | 30 |
| **Median age, y** | 51 |  | 45 | 14 | 31 | 51 | 43 | 61 | 46 | 30 |  | 59 | 52 | 45 | 52 | 14 |  |  |
| **% Obese** | 34 |  | 19 |  |  |  |  | 47 |  |  |  |  |  | 19 | 29 |  |  |  |
| **Age of inclusion, y** | ≥16 | ≥16 | ≥12 | 12-24 | All ages | ≥18 | All ages | ≥18 | ≥18 |  | ≥70 | ≥18 | ≥18 | ≥16 | ≥18 | 12-15 |  |  |
| **Timepoint**  **Since full**  **vaccination** | <7 d, 7 d- 60, 61 -120≥ 120 d | ≥7 d, ≥14 d | <7 d-36 d, 37-66 d, 67-96 d, 97-126 d, 127-156 d, ≥ 157 d | ≥14 d | 1,2,3,4,5,6, ≥7 d | <14 d, ≥14 d, 14 d-60 d,  61-120 d, ≥120 d | 0-6 d, 7-13 d, ≥ 14 d | >28 d | 0-3 d, 4-5 d, 7-13 d 14-39 d | 7-65 d | 0-3 d, 4-6 d, 7-13 d, ≥14 d | 14-120 d, >120 d | ≥ 1 d ≥ 8 d, ≥15 d | ≥7 days | ≥14 d, ≥28 d | ≥7 d | ≥14 d | ≥14 d |
| **Definition of severe infection** | FDA | WHO | WHO |  | WHO | Subjective* | WHO | WHO |  | WHO | WHO | WHO | WHO | WHO | ≥3 symptoms‡ | FDA |  |  |
| **Median follow-up**  **(mo)** | 6 | 2 | 3.6 | 1.9 | 7 | 6.1 |  | 2 | 1 | 2.6 |  | 4.7 | 3.7 | 0.5 | 1.9 |  |  |  |
| **Vaccine status determination** | Administered | Database | Database | Administered | Database | Administered | Database | Database | Database | Administered | Database | Database | Database | Database | Administered | Administered | Database | Database |
| **Quality score** | See Table S 2 | NOS Stars: 9 | NOS Stars:8 | See Table S 2 | NOS Stars: 8 | See Table S 2 | NOS Stars: 7 | NOS Stars: 8 | NOS Stars: 8 | NOS Stars: 6 | NOS Stars: 7 | NOS Stars: 8 | NOS Stars: 7 | NOS Stars: 8 | See Table S2 | See Table S2 | NOS Stars: 8 | NOS Stars: 7 |
| **Outcome measures** | Ab/NAAT/hx of COVID-19 | PCR | PCR | PCR | PCR | Ab | PCR | PCR | PCR/Ab | PCR | PCR | PCR/antigen | PCR | PCR | PCR |  | PCR | PCR |
| **Age subgroups, y** | <64, ≥65 | 16-44, 45-64, ≥65 | 12-15,  16-44,  45-64, ≥65 | 12-17,  18-25 | <60, ≥60 | 18-65, >65 |  |  |  |  |  | 18-49,  50-64,  ≥65 | 18-44,  45-64,  ≥65 | 16-39,  40-69,  ≥70 | 18-59, ≥60 |  |  |  |
| **Quality of evidence** | High | Moderate | Moderate | High | Moderate | High | Low | Low | Moderate | Moderate | Moderate | Low | Moderate | Moderate | High | High | Low | Moderate |

*Severe was defined as very bad symptoms, i.e., " I was not able to do activities that I usually do" or "I could not eat or drink" or "I have no taste or smell."

‡At least three signs or symptom and review from independent clinical severity adjudication committee.

† from first dose.

¶ blank cells indicate that information was not reported by that study.

Abbreviated: RCT: Randomized controlled trials; COVID-19 Coronavirus disease 2019; Ab: Antibody; WHO: World Health Organization; FDA: Food and Drug Administration; NOS: Newcastle-Ottawa Scale; US: United States of America; UK: United Kingdom; NAAT: Nucleic Acid Amplification Test. PCR: Polymerase chain reaction; ICU: Intensive care unit.; y: year; mo: months.

**Table S3:** Cochrane-risk-of-bias tool for randomized trials.

| Study | Thomas et al. 2021 | Ali et al. 2021 | Sahly et. al. 2021 | Sadoff et al 2021 | Frenck et al |
| --- | --- | --- | --- | --- | --- |
| Did the trial address a clearly focused issue? |  |  |  |  |  |
|  | yes | yes | yes | yes | yes |
| Was the assignment of patients to treatments randomized? |  |  |  |  |  |
|  | yes | yes | yes | yes | yes |
| Were all the patients who entered the trial properly accounted for at its conclusion? |  |  |  |  |  |
|  | yes | yes | yes | yes | yes |
| Were patients, health workers and study personnel 'blind' to treatment? |  |  |  |  |  |
|  | yes | yes | can't tell | yes | yes |
| Were groups similar at the start of the trial? |  |  |  |  |  |
|  | yes | yes | no | yes | yes |
| Aside from the experimental intervention, were the groups treated equally? |  |  |  |  |  |

**Figure S1:** **Forest plot of vaccine efficacy/effectiveness (VE) against SARS-CoV-2 infection stratified by vaccine type.** Mean VE values represent the mean VE against SARS-CoV-2 infection expressed as a percentage. Blue squares and their corresponding lines are the point estimates and 95% confidence intervals (95% CI). Maroon diamonds represent the pooled VE estimates for each subgroup (width denotes 95% CI). The p-value for interaction comparing the different subgroups <0.0001.

**Figure S2:** **Forest plot of vaccine efficacy/effectiveness (VE) against symptomatic COVID-19 stratified by vaccine type.** Mean VE values represent the mean VE against symptomatic COVID-19 expressed as a percentage. Blue squares and their corresponding lines are the point estimates and 95% confidence intervals (95% CI). Maroon diamonds represent the pooled VE estimates for each subgroup (width denotes 95% CI). The p-value for interaction comparing the different subgroups <0.0001.

**Figure S3:** **Forest plot of vaccine efficacy/effectiveness (VE) against severe COVID-19 stratified by vaccine type.** Mean VE values represent the mean VE expressed as a percentage. Blue squares and their corresponding lines are the point estimates and 95% confidence intervals (95% CI). Maroon diamonds represent the pooled VE estimates for each sub-group (width denotes 95% CI). The P-value for interaction comparing the different subgroups <0.0001.

**Figure S4:** **Forest plot of vaccine efficacy/effectiveness (VE) against SARS-CoV-2 infection stratified by RCT (efficacy trials) vs. non-RCT (effectiveness studies).** Mean VE values represent the mean VE expressed as a percentage. Blue squares and their corresponding lines are the point estimates and 95% confidence intervals (95% CI). Maroon diamonds represent the pooled estimates for each group (width denotes 95% CI). The p-value for interaction comparing the different subgroups <0.0001.

**Figure S5:** **Forest plot of vaccine efficacy/effectiveness (VE) against symptomatic COVID-19 stratified by RCT (efficacy trials) vs. non-RCT (effectiveness studies).** Mean VE values represent the mean VE expressed as a percentage. Blue squares and their corresponding lines are the point estimates and 95% confidence intervals (95% CI). Maroon diamonds represent the pooled estimates for each group (width denotes 95% CI). The p-value for interaction comparing the different subgroups <0.0001.

**Figure S6:** **Forest plot of vaccine efficacy/effectiveness (VE) against severe COVID-19 stratified by RCT (efficacy trials) vs. non-RCT (effectiveness studies).** Mean VE values represent the mean VE expressed as a percentage. Blue squares and their corresponding lines are the point estimates and 95% confidence intervals (95% CI). Maroon diamonds represent the pooled estimates for each group (width denotes 95% CI). The P-value for interaction comparing the different subgroups <0.0001.

**Figure S7:** **Forest plot of vaccine efficacy/effectiveness (VE) against SARS-CoV-2 infection stratified by WHO region.** Mean VE values represent the mean VE expressed as a percentage. Blue squares and their corresponding lines are the point estimates and 95% confidence intervals (95% CI). Maroon diamonds represent the pooled estimates for each group (width denotes 95% CI). The P-value for interaction comparing the different subgroups <0.0001. International group was represented by United States, Argentina, Brazil, South Africa, Germany, Turkey, Mexico, Colombia, Chile, and Peru.

**Figure S8:** **Forest plot of vaccine efficacy/effectiveness (VE) against symptomatic COVID-19 stratified by WHO region.** Mean VE values represent the mean VE expressed as a percentage. Blue squares and their corresponding lines are the point estimates and 95% confidence intervals (95% CI). Maroon diamonds represent the pooled estimates for each group (width denotes 95% CI). The P-value for interaction comparing the different subgroups <0.0001. International group was represented by United States, Argentina, Brazil, South Africa, Germany, Turkey, Mexico, Colombia, Chile, and Peru.

**Figure S9:** **Forest plot of vaccine efficacy/effectiveness (VE) against severe COVID-19 stratified by WHO region.** Mean VE values represent the mean VE expressed as a percentage. Blue squares and their corresponding lines are the point estimates and 95% confidence intervals (95% CI). Maroon diamonds represent the pooled estimates for each group (width denotes 95% CI). The P-value for interaction comparing the different subgroups <0.0001. International group was represented by United States, Argentina, Brazil, South Africa, Germany, Turkey, Mexico, Colombia, Chile, and Peru.


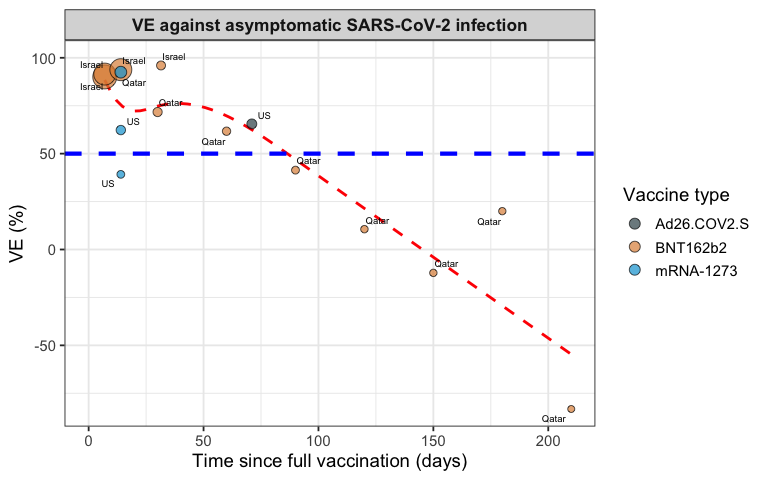


**Figure S10: Scatterplot and trend of vaccine efficacy/effectiveness (VE, %) against asymptomatic SARS-CoV-2 infection.** VE against asymptomatic SARS-CoV-2 infection (A) plotted according to time since complete vaccination (14 days after the second dose for mRNA vaccines and after one dose of vector-based vaccine). Each circle represents a study, and its size is proportional to the study’s sample size and annotated according to vaccine types. Trend red line is fitted by natural cubic splines. The horizontal blue line represents the 50% protection level stipulated by the WHO.[9]

A.

B.
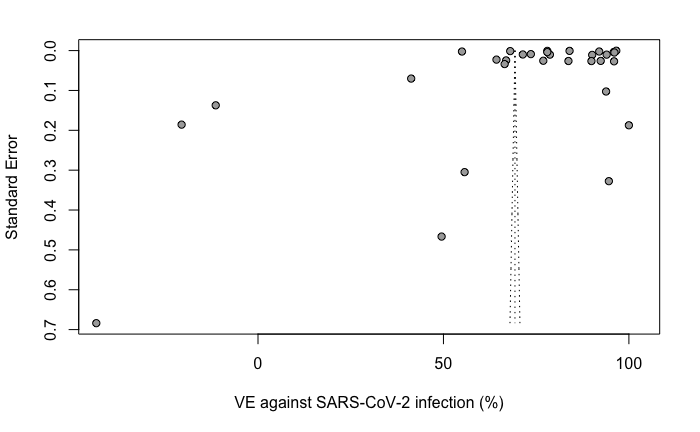


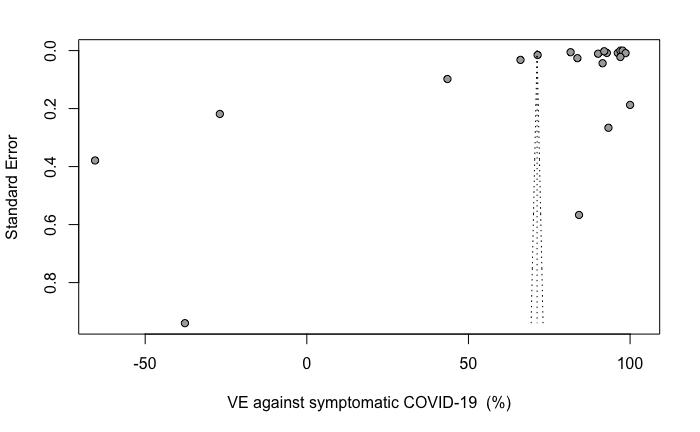


C.


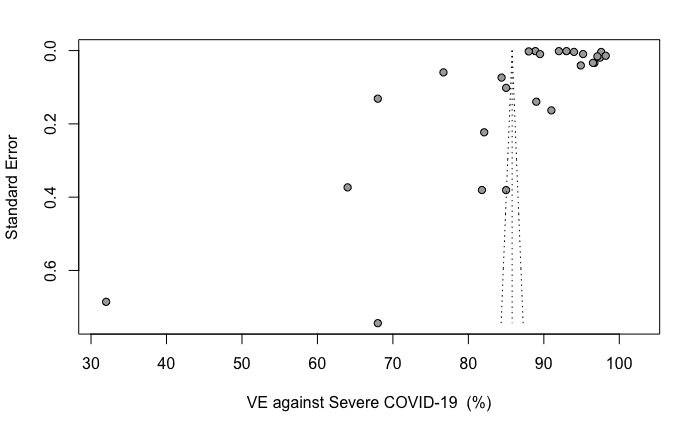


**Figure S11: Funnel plot for of vaccine efficacy/effectiveness (VE, %) against SARS-CoV-2 infection (A), symptomatic COVID-19 (B), and severe COVID-19 (C) included in the meta-analysis**. The shape of the funnel plots is uninterpretable.

**References**

1. Liu J, Liu Y, Xia H, Zou J, Weaver SC, Swanson KA, Cai H, Cutler M, Cooper D, Muik A: **BNT162b2-elicited neutralization of B. 1.617 and other SARS-CoV-2 variants**. *Nature* 2021, **596**(7871):273-275.

2. Liu Y, Liu J, Xia H, Zhang X, Zou J, Fontes-Garfias CR, Weaver SC, Swanson KA, Cai H, Sarkar R: **BNT162b2-Elicited Neutralization against New SARS-CoV-2 Spike Variants**. *New England Journal of Medicine* 2021.

3. Stowe J, Andrews N, Gower C, Gallagher E, Utsi L, Simmons R: **Effectiveness of COVID-19 vaccines against hospital admission with the Delta (B. 1.617. 2) variant**. *Public Health England* 2021.

4. Abu-Raddad LJ, Chemaitelly H, Butt AA: **Effectiveness of the BNT162b2 Covid-19 Vaccine against the B. 1.1. 7 and B. 1.351 Variants**. *New England Journal of Medicine* 2021.

5. Lopez Bernal J, Andrews N, Gower C, Gallagher E, Simmons R, Thelwall S, Stowe J, Tessier E, Groves N, Dabrera G: **Effectiveness of Covid-19 vaccines against the B. 1.617. 2 (Delta) variant**. *N Engl J Med* 2021:585-594.

6. Tenforde MW, Self WH, Naioti EA, Ginde AA, Douin DJ, Olson SM, Talbot HK, Casey JD, Mohr NM, Zepeski A: **Sustained effectiveness of Pfizer-BioNTech and Moderna vaccines against COVID-19 associated hospitalizations among adults—United States, March–July 2021**. *Morbidity and Mortality Weekly Report* 2021, **70**(34):1156.

7. Tartof SY, Slezak JM, Fischer H, Hong V, Ackerson BK, Ranasinghe ON, Frankland TB, Ogun OA, Zamparo JM, Gray S: **Effectiveness of mRNA BNT162b2 COVID-19 vaccine up to 6 months in a large integrated health system in the USA: a retrospective cohort study**. *The Lancet* 2021, **398**(10309):1407-1416.

8. Methley AM, Campbell S, Chew-Graham C, McNally R, Cheraghi-Sohi S: **PICO, PICOS and SPIDER: a comparison study of specificity and sensitivity in three search tools for qualitative systematic reviews**. *BMC health services research* 2014, **14**(1):1-10.

9. **Vaccine efficacy, effectiveness and protection** [<https://www.who.int/news-room/feature-stories/detail/vaccine-efficacy-effectiveness-and-protection>]

10. Higgins J, Altman DG: **Assessing risk of bias in included studies**. 2008.

11. Wells GA, Shea B, O’Connell D, Peterson J, Welch V, Losos M, Tugwell P: **The Newcastle-Ottawa Scale (NOS) for assessing the quality of nonrandomised studies in meta-analyses**. In*.*: Oxford; 2000.

12. DerSimonian R, Kacker R: **Random-effects model for meta-analysis of clinical trials: an update**. *Contemporary clinical trials* 2007, **28**(2):105-114.

13. Sidik K, Jonkman JN: **Robust variance estimation for random effects meta-analysis**. *Computational Statistics & Data Analysis* 2006, **50**(12):3681-3701.

14. IntHout J, Ioannidis JP, Borm GF: **The Hartung-Knapp-Sidik-Jonkman method for random effects meta-analysis is straightforward and considerably outperforms the standard DerSimonian-Laird method**. *BMC medical research methodology* 2014, **14**(1):1-12.

15. Higgins JP, Thompson SG, Deeks JJ, Altman DG: **Measuring inconsistency in meta-analyses**. *Bmj* 2003, **327**(7414):557-560.

16. Egger M, Smith GD, Schneider M, Minder C: **Bias in meta-analysis detected by a simple, graphical test**. *Bmj* 1997, **315**(7109):629-634.

17. Begg CB, Mazumdar M: **Operating characteristics of a rank correlation test for publication bias**. *Biometrics* 1994:1088-1101.

18. Gabbay F: **COVID-19: Developing Drugs and Biological Products for Treatment or Prevention-Background and New FDA Guidance**. 2020.
